# Is Chimeric Antigen Receptor T-cell Therapy the Future of Autoimmunity Management?

**DOI:** 10.7759/cureus.3407

**Published:** 2018-10-03

**Authors:** Amber Tahir

**Affiliations:** 1 Internal Medicine, Dow University of Health Sciences, Karachi, PAK

**Keywords:** chimeric antigen receptor, autoimmunity, car t-cell therapy, immune therapy, autoimmune diseases, allograft rejection

## Abstract

Clinical trials with chimeric antigen receptor (CAR) T-cell therapy in oncology have been promising. The spectrum of this novel therapy is being expanded to include autoimmunity. It ensures “targeted treatment,” resulting in more selective outcomes, fewer toxic effects, and a permanent restoration of immune system imbalance. The preliminary results of preclinical and clinical studies support the application of CAR therapy in autoimmunity, especially in regulating adverse autoimmune responses. This novel therapy should be considered for the treatment of autoimmune diseases and further clinical studies should be conducted to study all aspects of this treatment modality.

## Editorial

As the first two Food and Drug Administration (FDA)-approved CD-19 chimeric antigen receptor (CAR) T-cell products penetrated the United States market in 2017, the investment and interest in CAR therapy for hematological as well as solid malignancies has been expanding. With promising results from clinical trials and the success of the CAR-T therapy in oncology, clinicians and researchers developed an extensive interest in broadening the spectrum of CAR therapy applications beyond malignancies to include autoimmune diseases and allograft rejection [1-2].

In autoimmune diseases, there are either self-reactive or auto-antibodies produced by plasma cells or/and self-reactive T-lymphocytes. There can also be auto-reactivity against “sequestered antigens,” which are self-antigens already present in remote areas of the organism but not exposed in usual circumstances. These sequestered antigens become exposed to and suffer from auto-reactivity after an infection or lesion such as in multiple sclerosis. Cytokine imbalance, human leukocyte antigen (HLA) expression in target cells, reduced regulatory mechanisms, and defects in intrinsic T regulatory cells (Tregs) are also proposed mechanisms for autoimmunity [3].

CAR T-cell therapy has attracted autoimmunity researchers, as the therapy ensures “targeted treatment” as compared to the traditional, nonspecific treatment that revolves around the principal of immunosuppression/modulation and only weakly restores immune tolerance, hence requiring long-term treatment along with the risk of secondary infections and solid malignancies. Targeted monoclonal antibodies, including anti-TNFα, anti-CD19, and anti-IL6R, interact specifically with their targeted antigen, resulting in more selective outcomes, fewer secondary toxic effects, and a permanent restoration of the immune system imbalance. Although newer immunosuppressant/modulatory agents have been developed over time, which are more selective, focus only on localized targets, and have lower toxicity and side effects, they couldn’t contribute to a permanent restoration of immune system imbalance. Only CAR-T therapy has shown promising outcomes in this aspect.

A few interesting milestones have been achieved in treating autoimmune diseases by CAR-T therapy. For pemphigus vulgaris (PV) treatment, human T cells were engineered to express a chimeric autoantibody receptor (CAAR), which expressed PV autoantigen Dsg3. Dsg3-CAAR-T cells selectively targeted only the autoimmune B-cells because of their specificity for B-cell receptors (BCRs) [1]. The innovative benefits of this mode of treatment include decreased Dsg3 autoantibodies, specific cytolysis of autoreactive B-cells, long-term persistence of CAAR T-cells to ensure immunity, and no off-target toxic effects. This specific cytotoxicity of engineered CAAR-T cells against BCRs must be investigated further, as it has the potential to provide an efficient standard strategy for selectively targeting autoreactive B cells in antibody-mediated autoimmune disease. For the implication of CAR-T therapy in autoimmunity, the engineering of regulatory T cells (Tregs) has also been promising. Where in CAR-T therapy, the T cells attack their targets. Tregs are programmed to protect their target from being attacked by the immune system itself.

In another experiment, CAR-T therapy was implicated in hemophilia A patients to establish factor VIII immune tolerance [4]. Similar to CAARs, B-cell-targeting antibody receptors (BARs) were engineered. These BARs were transduced with human Tregs containing factor VIII C2 A2 domain. They targeted factor VIII-specific B-cells and deleted anti-factor VIII antibody production.

Nonclinical studies have shown Tregs to be a potential future for a variety of autoimmune disorders, including autoimmune hepatitis, inflammatory bowel disease, autoimmune encephalomyelitis, and arthritis [5]. CAR application beyond the spectrum of oncology seems favorable and will bring new energy to issues such as allograft rejection, multiple sclerosis, and systemic lupus erythematosus, where the current treatment options are getting exhausted and new options with promising outcomes are desperately needed. The preliminary results of preclinical as well as clinical studies support the application of CAR therapy in autoimmunity, especially in regulating adverse autoimmune responses and ensuring a permanent balance of the immune system. This novel therapy should be considered for the treatment of autoimmune diseases and further clinical studies should be conducted to study all aspects of this treatment modality.
